# Detection of colorectal cancer in urine using DNA methylation analysis

**DOI:** 10.1038/s41598-021-81900-6

**Published:** 2021-01-27

**Authors:** S. Bach, I. Paulis, N. R. Sluiter, M. Tibbesma, I. Martin, M. A. van de Wiel, J. B. Tuynman, I. Bahce, G. Kazemier, R. D. M. Steenbergen

**Affiliations:** 1 Department of Surgery, Cancer Center Amsterdam, Amsterdam UMC, Vrije Universiteit Amsterdam, De Boelelaan 1117, Amsterdam, The Netherlands; 2 Department of Pathology, Cancer Center Amsterdam, Amsterdam UMC, Vrije Universiteit Amsterdam, De Boelelaan 1117, Amsterdam, The Netherlands; 3 Department of Epidemiology and Data Science, Amsterdam Public Health Research Institute, Amsterdam UMC, Vrije Universiteit Amsterdam, De Boelelaan 1089a, Amsterdam, The Netherlands; 4 Department of Pulmonary Diseases, Cancer Center Amsterdam, Amsterdam UMC, Vrije Universiteit Amsterdam, De Boelelaan 1117, Amsterdam, The Netherlands

**Keywords:** Gastrointestinal cancer, Tumour biomarkers

## Abstract

Colorectal cancer (CRC) is the second leading cause for cancer-related death globally. Clinically, there is an urgent need for non-invasive CRC detection. This study assessed the feasibility of CRC detection by analysis of tumor-derived methylated DNA fragments in urine. Urine samples, including both unfractioned and supernatant urine fractions, of 92 CRC patients and 63 healthy volunteers were analyzed for DNA methylation levels of 6 CRC-associated markers (*SEPT9*, *TMEFF2*, *SDC2*, *NDRG4*, *VIM* and *ALX4*). Optimal marker panels were determined by two statistical approaches. Methylation levels of *SEPT9* were significantly increased in urine supernatant of CRC patients compared to controls (*p* < 0.0001). Methylation analysis in unfractioned urine appeared inaccurate. Following multivariate logistic regression and classification and regression tree analysis, a marker panel consisting of *SEPT9* and *SDC2* was able to detect up to 70% of CRC cases in urine supernatant at 86% specificity. First evidence is provided for CRC detection in urine by *SEPT9* methylation analysis, which combined with *SDC2* allows for an optimal differentiation between CRC patients and controls. Urine therefore provides a promising liquid biopsy for non-invasive CRC detection.

## Introduction

Colorectal cancer (CRC) is the third most commonly diagnosed cancer worldwide and the second leading cause for cancer-related death, accounting for 10–12% of all cancer cases^1, 2^. Currently, there is a high clinical need for a non-invasive CRC biomarker test. Ideally the biomarker should have high accuracy and be able to provide means for detection of CRC, prognostication and disease monitoring during and after treatment^3^. Due to the absence of symptoms during early stages of disease, nearly half of patients are diagnosed at an advanced stage. Cancer stage at diagnosis however strongly correlates with survival, illustrated by the 5-year survival rate of 95% for stage I as compared to only 11% for stage IV^2^. Timely detection of CRC could improve mortality rates due to better chances of diagnosing CRC at a more curable disease stage^4^. Screening for CRC is therefore recommended in the western world as it is helps to lower the death rate due to CRC^5^. Screening tests are stool-based or visual. However, both are cumbersome and colonoscopy carries risks as it is invasive^6, 7^. The ongoing development and increasing palette of CRC treatments require better tools to prognosticate patients. As molecular subtyping is key, colonoscopies and invasive biopsies are often warranted to decide on the appropriate treatment strategy^8, 9^. Furthermore, more accurate disease monitoring and detection of minimal residual disease following treatment is necessary. Existing tools, including imaging and engrained protein-based biomarker CEA, lack sensitivity or come with risks. Non-invasive means for accurate detection of a molecular signature of CRC would mean opportunities for each of the early detection, prognostication and monitoring modalities. This urgent need has led to extensive research on molecular CRC biomarkers in recent years, with growing interest in liquid biopsies.

A ‘liquid biopsy’ enables the analysis of cancer derived molecules in biofluids, including peripheral blood, saliva and urine^10^. Blood containing cell free DNA (cfDNA) is the most studied liquid biopsy to date. Cell free DNA consists of extracellular nucleic acids released into the circulation by means of necrosis, apoptosis and active secretion^11^. A small fraction of the total cfDNA consists of tumor derived DNA, so called circulating tumor DNA (ctDNA). Detection of ctDNA can be facilitated by analysis of DNA methylation, which is the enzymatically induced covalent binding of a methyl group (-CH_3_) to cytosine-guanine dinucleotides (CpG)^12^. It is a common epigenetic regulator of gene expression, frequently altered in cancer^13^. Increased methylation in CpG dense regions, called CpG islands and located in promoter regions of tumor suppressor genes, can lead to inactivation of tumor suppressor genes. As this is believed to be an early and critical event in CRC development, analysis of ctDNA methylation has potential to serve as a biomarker for CRC^3, 14–16^.

Circulating tumor DNA is excreted in urine through glomerular filtration, which propagates urine analysis as a true non-invasive method of cancer detection^17–19^. Despite advances in ctDNA diagnostics in blood, the process of venipuncture remains invasive and many challenges still have to be tackled. Analysis of ctDNA in urine features a non-invasive and logistically attractive way of testing.

Urinary cfDNA can roughly be divided into two groups based on fragment size, comprising high- and low molecular weight (MW) groups. The high-MW group consists of heterogeneous DNA fragments of 1 kilobasepair (kbp) and larger, typically originating from the cell debris of the urogenital tract^20, 21^. The low-MW group consists of smaller DNA fragments between 150 and 250 bp. Presumably, the low-MW fraction of urinary DNA is partially derived from the blood circulation, allowing detection of ctDNA in urine samples^21, 22^. One method by which urine samples can be enriched for low-MW DNA is by centrifugation, which partly separates potential tumor DNA from non-specific high-MW DNA^18^. In contrast to, for example bladder cancer, in which high-MW DNA present in urinary sediment is suitable for tumor DNA detection^23^, the supernatant fraction is expected to be of most interest for detection of non-urogenital tumors, such as colon cancer, in urine.

In this study, we aimed to evaluate the diagnostic potential of urine DNA methylation analysis for detection of CRC.

## Material and methods

### Study subjects and sample processing

Consecutive patients suffering from CRC who visited the Surgery Department Amsterdam UMC, a tertiary referral center in, Amsterdam, The Netherlands between January 2018 and February 2019, were included in the study. All patients were older than 18 years, were diagnosed with a pathology proven CRC, and underwent no recent anticancer treatment during the last year. Patients with other malignancies in the previous 3 years were excluded. All participating patients provided urine samples during visits to the outpatient clinic prior to surgery. Samples were collected in 40 ml containers containing 40 mM Ethylenediaminetetraacetic acid (EDTA) and subsequently processed within 6 h and stored at 4 °C. Both addition of EDTA and storage at 4 °C preserves urine DNA for accurate methylation analysis^24^. Healthy volunteers, serving as controls, were selected for eligibility through a pre-defined selection process. By taking a questionnaire, it was verified if control subjects were not diagnosed with cancer at any point during their life, and matched the age range of the CRC patient test groups. Urine samples from controls were also collected in containers containing 40 mM EDTA and processed upon arrival. For both CRC patients and controls an independent set of consecutively collected urine samples was used for our studies on unfractioned urine and urine supernatant. To obtain the urine supernatant fraction and enrich for low-MW, DNA samples were centrifuged at 3000 g for 15 min. Unfractioned and supernatant urine specimens were frozen at − 20 °C until further use.

Sample collection and study design were approved by the Medical Ethical Committee board of the Amsterdam UMC for both CRC patients and healthy volunteers (no. 2018.035 and no. 2018.657). Written informed consent was obtained from all participants of this study. All experiments were performed in accordance with relevant guidelines and regulations.

### DNA isolation and bisulfite modification

For DNA isolation from unfractioned urine and urine supernatant the Quick DNA urine kit (Zymo Research, Irvine, CA, US) was used. This isolation method proved superior for the isolation of small DNA fragments (as low as 50 bp) over other DNA isolation methods (data not shown). DNA from the CRC cell line RKO (American Type Culture Collection) was isolated using the PureLink genomic DNA kit (Invitrogen, Waltham, MA, US).

Isolated DNA was eluted in 50ul elution buffer. DNA concentrations were measured using the Qubit™ dsDNA HS Assay (Invitrogen, Carlsbad, CA, US). For methylation analysis, up to 400 ng of isolated DNA was treated with bisulfite using the EZ DNA Methylation kit (Zymo Research, Irvine, CA, US). All procedures were performed according to manufacturer’s guidelines.

### Quantitative methylation specific PCR (qMSP)

Six CRC- associated DNA methylation markers (*SEPT9*, *TMEFF2*, *SDC2* and *NDRG4*, *VIM* and *ALX4)* were selected from a systematic literature search based on accuracy^3^.

Two multiplex quantitative Methylation Specific PCRs (qMSPs), each consisting of 3 targets (*SEPT9, TMEFF2 & SDC2* and *NDRG4, VIM,& ALX4*) and reference gene (β-actin: ACTB) were designed based on sequences as described previously^25–29^. By adjusting amplicon sizes to a maximum of 80 bp, detection of CRC-derived low-MW DNA was facilitated. Multiplex development was executed according to optimization parameters as described by Snellenberg et al*.*^30^*.* In brief, marker specificity was individually evaluated by MSP with unmodified and modified DNA isolated from CRC cell line RKO. Sensitivity analysis demonstrated that both qMSP multiplexes were able to detect methylated RKO DNA diluted in water up to dilutions of 0.1% and 0.5%, respectively. Primer and probe limiting assays were performed to determine their ideal concentrations in both singleplex and multiplex qMSP’s. qMSP analysis was performed on a ViiA7 real-time PCR-system (ThermoFisher Scientific, Waltham, MA, USA), using Epitect Multiplex PCR Mastermix (Qiagen, Venlo, Netherlands) Methylation marker abundance was calculated relative to ACTB levels (Ct-ratio), using the following formula: 2 − (CtMARKER − CtACTB) * 100. Further details are provided in supplementary S1.

### Data analysis

Ct-ratios of methylation targets were compared between groups using the Mann Whitney U test. Results from statistical tests were corrected for multiple testing by the Bonferroni-procedure. Differences in absolute detection rates were compared and tested for statistical significance with the Pearson’s Chi-square test. *p* values < 0.05, adjusted using Bonferroni correction, were considered to be statistically significant.

Analyses of relationships between methylation and clinical parameters were only performed for marker *SEPT9*, since all other markers did not have sufficient data points for additional statistical analysis. In the patient group, *SEPT9* was compared to cancer stage by the Kruskal Wallis test. Due a large proportion of stage IV patients in our study having only peritoneal metastases (68%), results of stage IV patients were split up between peritoneal metastasis solely and stage IV including all types of (including hematogenous) metastasis. In this study these were only liver metastases.

To determine the ability of a combination of markers to differentiate between controls and patients, two approaches were explored for determining both the best marker panel and marker thresholds for a maximal test accuracy. In the first method, multivariate logistics regression (MLR) was used to model the probability of a urine sample being from a CRC patient, with all six methylation markers as independent variables. First, we fit a model with the six main effects only, and selected markers by stepwise selection. Then, to investigate whether the in-model effect of an individual marker was affected by other markers, we added the two-way interaction terms that include the selected main effects, again followed by stepwise selection. A leave-one-out cross-validation was then used to evaluate the performance of the model for prediction. Next, the predicted probability from this cross-validation was used for sample classification, according to a maximal Youden’s index, i.e. the sum of sensitivity and specificity minus 1. For fitting the MLR model, the R function Generalized Linear Models or glm was used. Apart from the MLR, we applied an algorithm-based method called classification and regression tree (CART) for binary classification of cases and controls on the same set of methylation markers. For this alternative analysis, a decision tree was obtained allowing for classification of urine samples based on marker values. We refer to^31^ for further details on CART method. For the purpose of prediction, the predicted class was obtained by leave-one-out cross validation. For both building the decision tree as well as performing prediction, the R package Recursive Partitioning or rpart was used.

The performance of both methods was determined from obtained sensitivities and specificities. For logistics regression, the Receiver Operator Characteristic (ROC) curve was plotted together with maximized Youden’s index.

Statistical analyses were performed using SPSS software (SPSS 22.0, IBM, Armonk, NY, USA) and R (Vienna, Austria. UR). Data visualization and construction of graphs was facilitated by GraphPad (Graphpad Prism version 8.2.1, La Jolla, CA USA). Additional details of all statistical analyses can be found in supplementary file S1.

## Results

### Patient and sample characteristics

In total 47 CRC patients and 20 healthy controls were included in the unfractioned group, and 45 CRC patients and 43 controls in the supernatant group. Clinical characteristics of CRC patients and controls with valid qMSP results are depicted in Table 1.

**Table 1:** Baseline and disease characteristics of CRC patients and controls.

Characteristic	*Unfractioned urine*	
	Colorectal cancer (n = 47)	No malignancy (n = 20)
**Sex**		
Male	28 (60%)	10 (50%)
Female	19 (40%)	10 (50%)
**Age**		
Median years (IQR)	66 (60–76)	65 (61–69)
**Stage**		
1	8 (17%)	
2	12(26%)	
3	11 (23%)	
4	16 (34%)	

Characteristic	*Urine supernatant*	
	Colorectal cancer (n = 45)	No malignancy (n = 43)
**Sex**		
Male	25 (56%)	13 (30%)
Female	20 (44%)	30 (70%)
**Age**		
Median years (IQR)	66 (59- 74)	60 (53–67)
**Stage**		
1	8 (18%)	
2	9 (20%)	
3	8 (18%)	
4	20 (44%)	
**Stage IV characteristics**		
Primary present	10 (50%)	
Liver metastases	5 (75%)	
Peritoneal metastases	15 (25%)	

The DNA yield of both unfractioned urine and urine supernatant collected from CRC patients and controls were evaluated to assess the utility for methylation analysis. The sample DNA concentrations are shown in Table 2. Concentrations of unfractioned urine samples were approximately three to five times higher as compared to supernatant samples, for patients and controls. Regarding sex, unfractioned urine DNA concentrations of female subjects were two times higher than DNA concentrations measured in male subjects.

**Table 2:** Median DNA concentrations of samples.

ng DNA per ml urine			
Unfractioned urine	Male	Female	Total
CRC (min–max)	7.4 (0.3–112.5)	16.1 (1.3–142.5)	10.9 (0.3–142.5)
Control (min–max)	14.7 (1.3–142.5)	27.0 (1.1–114.5)	22.2 (1.1–114.5)

Supernatant	Male	Female	Total
CRC (min–max)	2.2 (0.2–147.5)	2.7 (0.8–142.5)	2.2 (0.2–147.5)
Control (min–max)	3.3 (0.1–133.8)	8.0 (1.0–168.8)	6.0 (0.1–168.8)

### DNA methylation detection rates in unfractioned urines samples

**Figure 1:**
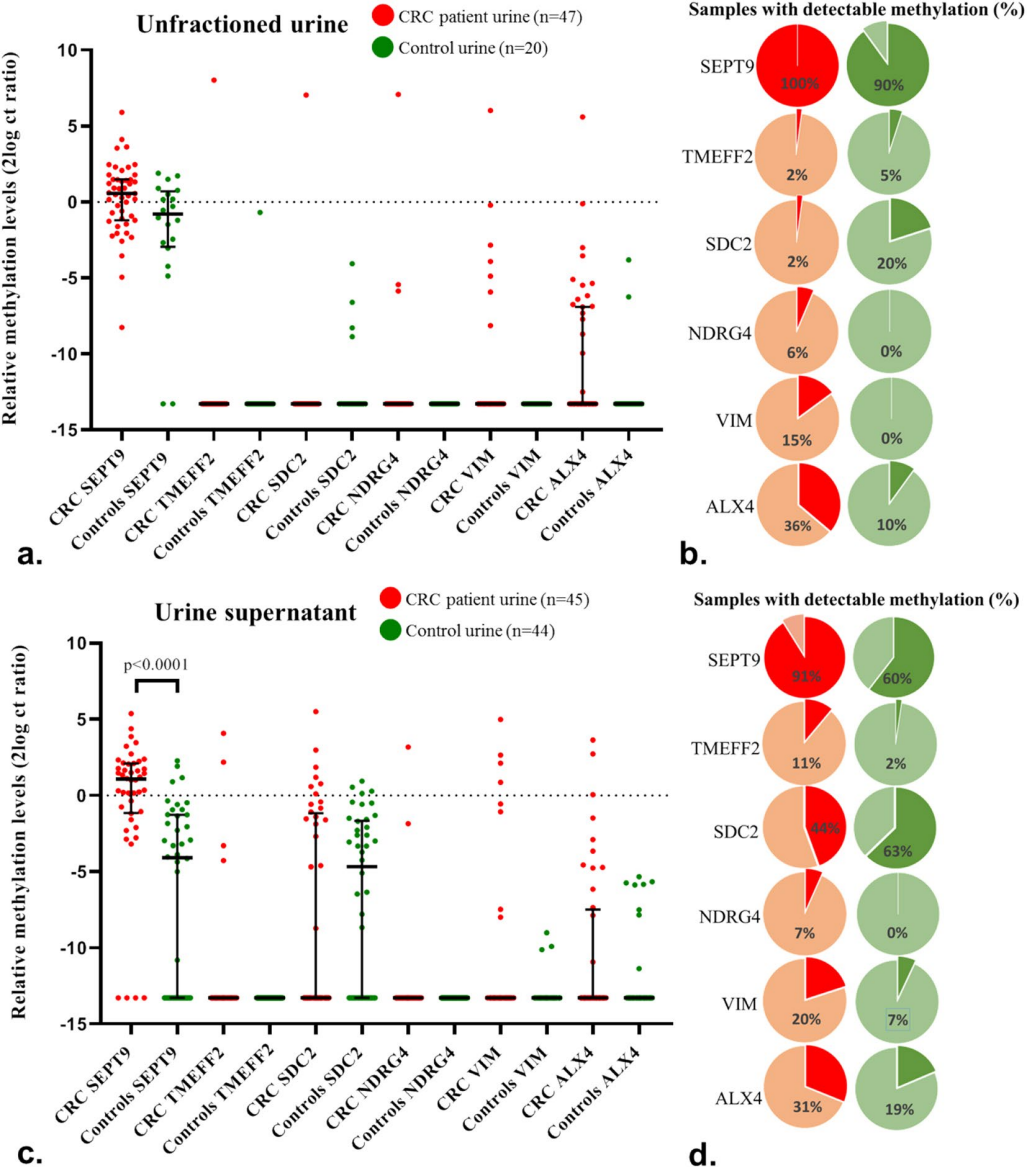
Methylation levels (a + c) and detection rates (b + d) in both unfractioned urine and urine supernatant samples. (**a**, **c**) Shows methylation detection of *SEPT9, TMEFF2, SDC2, NDRG4, VIM and ALX4* in unfractioned and supernatant urine samples of CRC patients and controls. Data are shown as the median with interquartile range of 2log converted Ct ratios. In  **b**, **c**, detection rates of markers in unfractioned urines and supernatant samples of CRC patients and controls are depicted, meaning the percentage of samples scoring a CtMARKER value below 45.

DNA methylation of *SEPT9, TMEFF2, SDC2, NDRG4, VIM* and *ALX4* in unfractioned urine samples of CRC patients (n = 47) and controls (n = 20) was investigated to evaluate their potential for CRC detection. Elevated methylation levels were detected in a subset of CRC patients for *SEPT9*, and at low frequencies for *VIM* and *ALX4* (Fig. 1a). Following Bonferroni correction, none of the markers was found to be significantly different between patients and controls. Likewise, no significant differences were seen for methylation detection rates, defined as any positive signal in qMSP analysis (i.e. Ct value < 45)(Fig. 1b). Methylation marker *SEPT9* was detected in all CRC patients as well as nearly all controls (90%). The remaining markers were detectable in 2–36% of CRC patients and 0–20% of controls.

### DNA methylation detection rates in urine supernatant samples

Next we determined if the supernatant fraction, which is presumed to be enriched for cfDNA^18^, would allow for a better discrimination between patients and controls. The same methylation markers were tested on urine supernatants from an independent cohort of CRC patients (n = 45) and controls (n = 44). As shown in Fig. 1c, *SEPT9* methylation levels were significantly elevated in CRC patients compared to controls (*p* < 0.0001). No significant differences were found for the remaining five markers. Assessment of the absolute detections rates also demonstrated that *SEPT9* methylation analysis detected significantly more CRC patients than controls (*p* < 0.01)(Fig. 1d). No differences in detection rates between the two groups were found for the other five markers.

**Figure 2:**
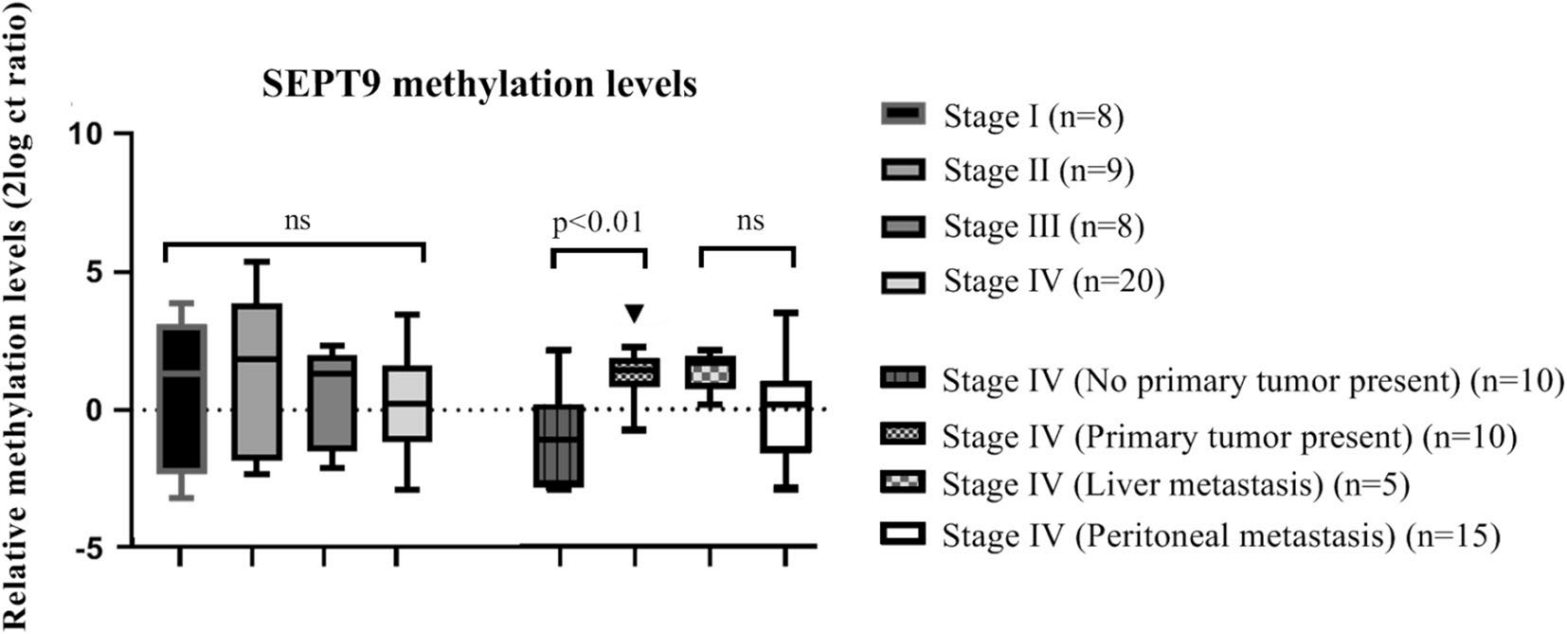
*SEPT9* methylation levels in urine supernatant per CRC stage. Samples of patient with stage IV CRC have been further stratified in patients with and without the primary tumor still being present. Furthermore, methylation levels in patients with solely intraperitoneal metastases (n = 15) and patients diagnosed with stage IV due to liver metastases (n = 5) are shown. Data are shown as the median with interquartile range of 2log converted Ct ratios.

In the group of CRC patients, no difference in *SEPT9* methylation levels was found between the different clinical stages of CRC disease (Fig. 2). However, within the stage IV patients, *SEPT9* methylation levels were significantly increased in patients of which the primary tumor was still present during urine collection, compared to stage IV patients having a history of resection of the primary CRC tumor (*p* < 0.01). Patients with solely peritoneal metastases had a trend towards lower levels of urine ctDNA, as compared to patients with liver metastases.

### Discriminating potential of combined methylation markers in urine supernatant

The diagnostic potential of a combined panel of DNA methylation markers was evaluated to differentiate between healthy controls and CRC patients. Both a multivariate logistics regression (MLR) and classification and regression tree (CART) analysis methods were used to assess accuracy. Furthermore, discrepancies were determined between models with regard to sample classification. To allow for a complete analysis, CRC samples (n = 2) and control samples (n = 1) that had an invalid ACTB in one of the two multiplexes, were discarded in this process which resulted in a total of 43 CRC and 42 control urine supernatants to be evaluated.

When fitting the MLR model with main effects, only *SEPT9* was significantly associated with the probability of being in the case group (*p* < 0.0001). Therefore, a logistics regression was fitted, with *SEPT9* and the interaction terms with the other five markers. The stepwise selection procedures selected *SEPT9* methylation and the interaction term between *SEPT9* and *SDC2* methylation to be strongly associated with the probability of being a CRC patient. Figure S2 illustrates the behavior of the estimated probability of being a CRC case for various values of *SEPT9* and *SDC2*. In this model, when methylation levels of *SEPT9* were low, the probability of being a case was small, irrespective of *SDC2* levels. In the higher values of *SEPT9* however, we noticed that gradual increases of predicted probabilities of being a case were affected by the values of *SDC2*. This explains the interactive effect of *SEPT9* and *SDC2*.

**Figure 3:**
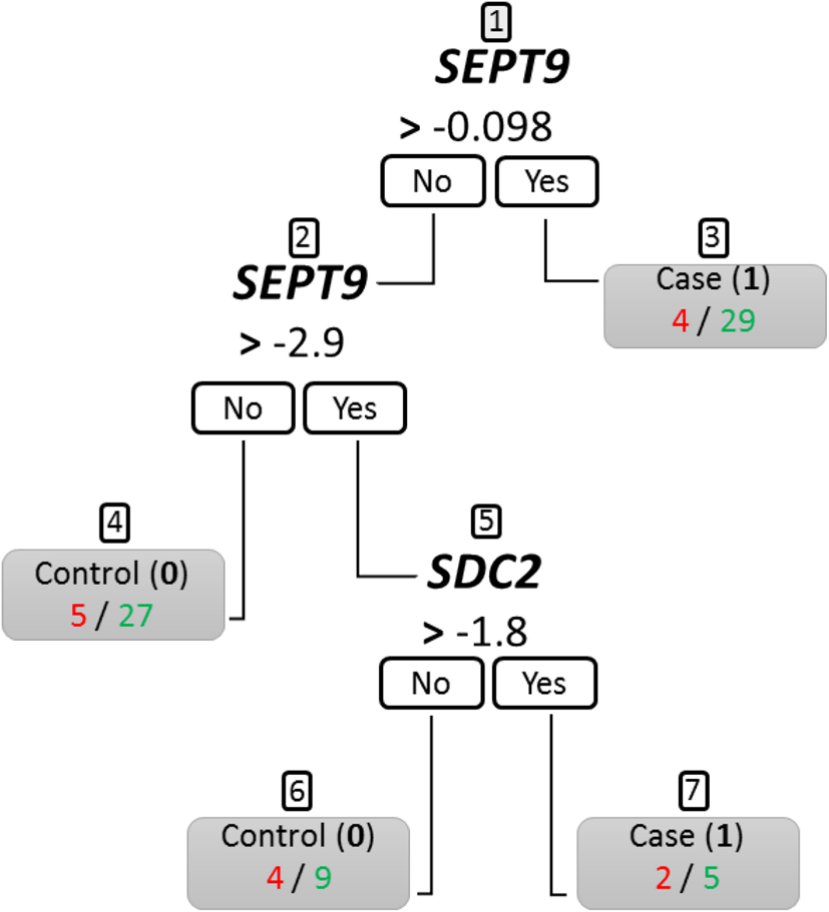
The CART decision tree. The boxes depict the decision nodes. Based on *SEPT9* or *SDC2* methylation values, samples are classified as a case (1) or control (0). The numbers below the node represent urine samples that are classified incorrectly (red) or correctly (green) according to the classification of 0 or 1. Node numbers are indicated above by 1 to 7.

In the second approach, we used a CART model to classify cases and controls based on the values of each markers. The resulting decision tree is depicted in Fig. 3. As in the logistics regression, *SEPT9* is the most important predictor for the classification, but again *SDC2* constitutes an interaction variable. When *SEPT9* methylation was higher or equal to -0.098, subjects were classified into cases (Fig. 3, node 3). In node 3, there are 29 correctly classified and 4 misclassified subjects. In the next step, subjects were classified into controls when having *SEPT9* methylation lower than -2.9. In this branch, 27 subjects were correctly classified and 5 subjects were misclassified (node 4). Finally, when the value of *SEPT9* was lower than -2.9, the threshold of *SDC2* = − 1.8 determined the controls (i.e. *SDC2* ≤ − 1.8, with 9 correctly classified and 4 misclassified subjects) and cases (i.e. *SDC2* > − 1.8, with 5 correctly and 2 misclassified subjects).

**Figure 4:**
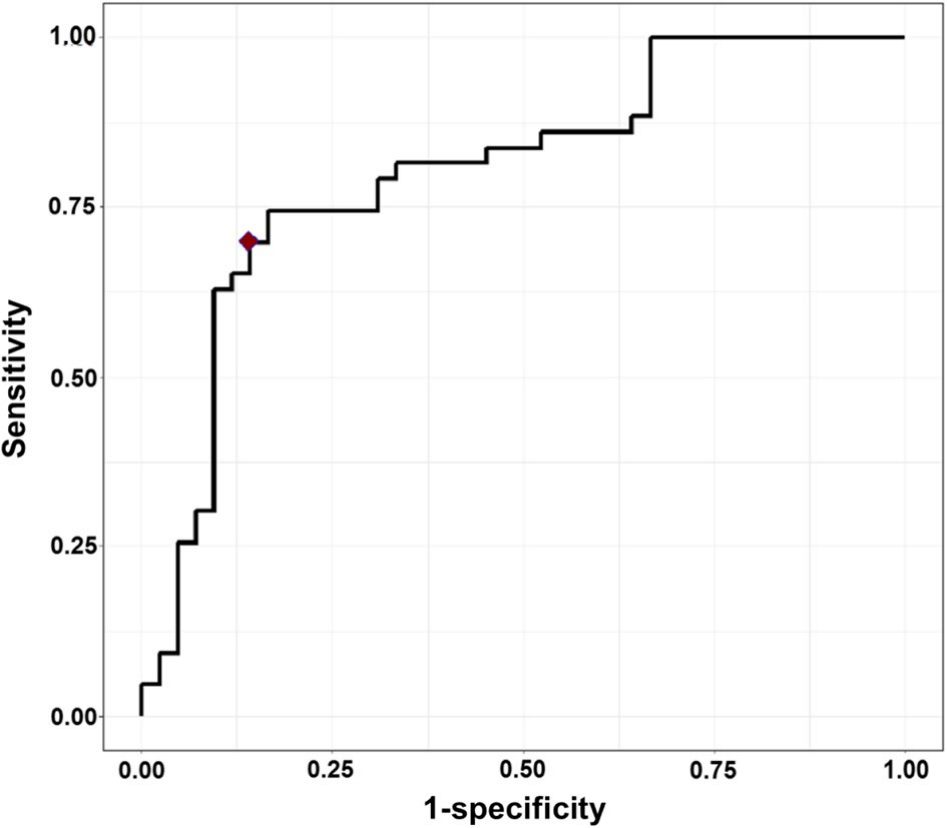
The ROC curve for classification of supernatant urine samples using multivariate logistics regression (MLR) model with marker *SEPT9* and interaction of markers *SEPT9* and *SDC2*. The red diamond indicates the maximal Youden’s index.

**Figure 5:**
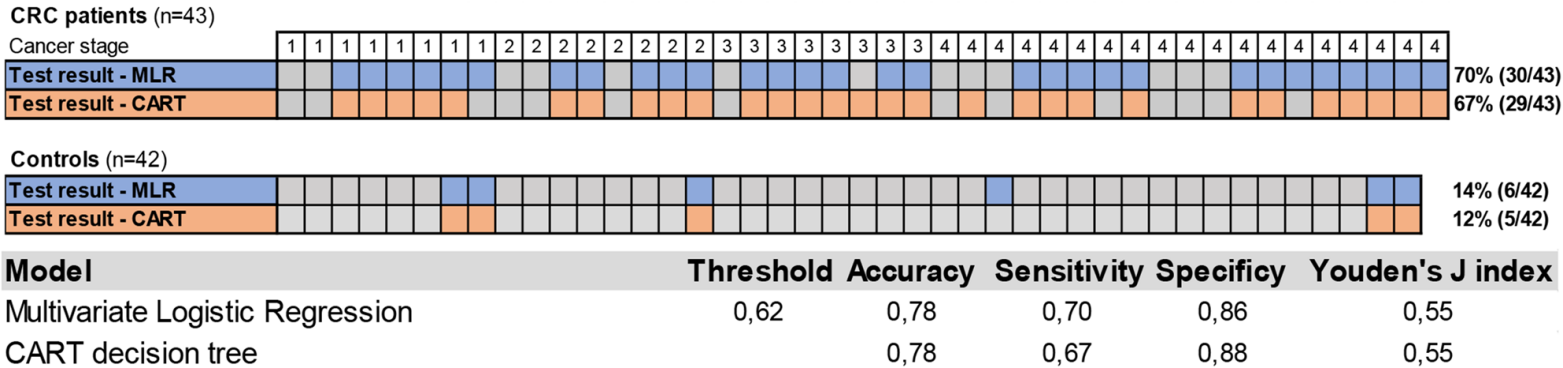
The performance of both generated models and their accuracy in detection of CRC in supernatant urine. The figure shows the classification of CRC samples and controls for both MLR and CART models. At the bottom, performance specifics for both models are depicted.

Finally, leave-one-out cross validation was utilized to evaluate the prediction performance of MLR and CART. Using the MLR model, we obtained the estimated probability of being a case while the CART decision tree assigns a sample as case or control (Figure S4). Hence, unlike CART decision tree, logistic regression allowed drawing a receiver operating characteristic (ROC)-curve (Fig. 4). A maximized Youden’s index was used to compare the performances of both methods. Figure 5 illustrates the performance of both models. In general, the performance of both MLR with interaction and CART were almost similar. While the MLR provided slightly higher sensitivity compared to CART (70% vs 67%), the latter had a slightly better specificity (88% vs 86%). Furthermore, the two models agreed on the classification of > 90% of all samples (Fig. 5). Both models were also able to detect CRC independent of cancer stage.

## Discussion

This study demonstrates for the first time that urine of CRC patients contains elevated levels of the DNA methylation marker *SEPT9*, as compared to healthy control patients. *SEPT9* methylation, combined with marker *SDC2*, offers a potential novel tool for detection and monitoring of CRC. Using short-amplicon methylation specific PCRs, we have successfully detected CRC-associated DNA methylation in urine supernatant. Out of six markers tested, *SEPT9* showed best accuracy to serve as a potential urinary biomarker for CRC detection. By combining *SEPT9* and *SDC2*, up to 70% of CRC cases could be detected at a specificity of 86%.

Despite extensive research, a need still exists for a non-invasive biomarker to detect CRC during clinical management. While many studies have been performed on the use of ctDNA in plasma for these purposes, a possible role for urine has not yet been well elucidated. Urine as a biofluid poses several advantages over blood. It does not require trained professionals to acquire, it lends itself for easy repeated sampling and it has been shown that urine poses a very stable medium for DNA^24^. This allows for reliable testing of samples collected in an ambulant setting. Additionally, there are no limits to available quantities. For screening programs of other types of cancer, urine is currently evaluated as a non-invasive alternative to physician-involved diagnostics^32–35^. The results from the present study suggest that urine has the same potential for CRC detection, by showing ctDNA is detectable through means of DNA methylation analysis.

This report is among the first few publications exploring the feasibility of molecular analysis in urine for the purpose of non-invasive CRC detection. In a pioneer study by Su et al., the distinction between high and low-molecular weight (HMW and LMW) urine DNA for CRC detection was made, showing the latter provides higher accuracy for detection of CRC-specific *KRAS* DNA mutations^22^. Some methylation markers tested in the present study have been described before for CRC detection in urine samples. Methylation marker *VIM* was assessed in two separate studies^28, 36^. In a study that selected low molecular weight DNA from urine samples using carboxylated magnetic beads, 12 of 17 LMW (71%) urine DNA samples of CRC patients were found to be positive for *VIM* methylation, compared to two out of 20 (10%) control samples^28^. Another earlier publication however, showed a poor performance of *VIM* methylation detection in urine (i.e. 8% sensitivity at 100% specificity). This study also assessed the methylation markers *WIF-1* and *ALX4* in urine, for which respectively a sensitivity of 27% and 15% at 99% and 100% specificity was found^36^. Detection of *NDRG4* methylation in urine has been described earlier by Xiao et al*.* with 55 of 76 (73%) CRC cases testing positive for urine methylation, at a specificity of 85% based on 36 controls^29^.

Differences in methodology or sample population may explain the discrepancies in CRC detection rates of methylation markers *NDRG4* and *VIM*. In the present study, centrifugation appeared effective for enrichment of highly fragmented tumor DNA (low-MW), as supernatant samples enabled a more adequate differentiation between disease and healthy controls compared to unfractioned urine that contains both high-MW and low-MW DNA. Furthermore, we used a dedicated urine DNA isolation kit. Interestingly, all mentioned previous studies did not fractionate urine samples prior to DNA isolation and did not use specialized urine DNA kits. Song et al*.* stored urine samples on − 70 °C directly following collection, and isolated for low-MW DNA using magnetic beads-based selection and resin-based DNA isolation after defrosting of the urine samples^28^. Xiao et al. isolated DNA directly following collection, however no pre-PCR DNA size selection was performed and no information was given on the isolation method^29^. Amiot et al*.* did not provide any details with regard to urine processing and used the same generic DNA isolation kit for urine, plasma and stool^36^. Concerning differences in sample population, Song et al. collected control samples from patients that underwent a colonoscopy yielding negative results. The other studies did not provide further information on their controls. Furthermore, differences in test group ethnicity might have influenced baseline methylation levels^37^. Another important reason of discrepancies in marker performance between present and earlier studies, could be differences in the actual targeted marker CpG dinucleotides undergoing PCR amplification.

Besides limited data on the use of urine for detection of CRC, little evidence is currently available for prognostication and disease monitoring. By detection of *KRAS* mutations in urine, only Fuji et al*.* explored the modality of advanced CRC treatment monitoring*,* showing a decline of urinal/urinary *KRAS* mutations during effective systemic therapy^38^.

To our knowledge, this is the first study describing detection of *SEPT9* methylation in urine. Urine *SEPT9* methylation analysis showed a sensitivity for CRC detection coming close to those reported for *SEPT9* methylation analysis in plasma samples, which vary from 75 to 81% (at ≥ 96% specificity)^39^. *SEPT9* methylation analysis for CRC detection in plasma is now available as an FDA-approved commercial test (Epi ProColon 2.0, Epigenomics AG Coporation, Berlin, Germany). Established CRC plasma methylation markers tested in this study, other than *SEPT9*, showed lower detection rates. Possible explanations include both biological and technical causes. Urine as a biofluid might have properties that lead to decreased detection of certain ctDNA fragments. Pores present in the glomerular basal membrane (GBM) may select not only on molecular weight, but also its net negative electric charge could play a role in preventing blood-urine translocation of the negatively charged DNA^19, 40^. Other thermodynamic properties of DNA that lead to its polymorphic potential, as well as complex formation with for instance proteins, might also influence the probability of glomerular translocation. As these properties are hugely influenced by particular nucleotide sequences of ctDNA, certain methylation markers might have a decreased performance in urine. A comparison between blood and urine samples from the same test subjects would therefore be very interesting to estimate the rate by which the methylation signal gets lost due to GBM filtration.

No differences were found between urine *SEPT9* methylation levels and clinical stages of CRC of patients. Although the detection of ctDNA appears more likely when sampling occurs during higher clinical stages of neoplastic disease, this was not the case in the present study. Similarly, the study on *VIM* methylation in CRC urine samples did also find no correlation with CRC stages^28^. For stage IV patients however, we found that the presence of the primary tumor may be an attributive factor for detecting CRC-associated methylation in urine DNA. This probably also relates to the fact that the majority of included stage IV patients without the primary tumor present were suffering from peritoneal metastases. Data of our group shows that patients with peritoneal metastases have a smaller tendency to have detectable ctDNA^41^. Furthermore, the majority of peritoneal metastases are classified as CMS4 (Consensus Molecular Subtypes). In this tumor subtype, methylation levels are often very low^42, 43^.

A novel approach in this study is the use of two different methods of statistical analysis to determine the complementarity between methylation markers to achieve maximal accuracy for CRC detection. The MLR and CART models agreed on classification of most urine samples, supporting the validity of our results. The MLR model gave a slightly higher sensitivity (70%) compared to the decision tree (67%), whereas the CART model yielded a slightly higher specificity (MLR: 86%, CART: 88%). In this study, the MLR model was optimized to achieve a maximal Youden’s index but could, depending on the clinical context, be adjusted to achieve a higher sensitivity or specificity. Furthermore, it should be noted that especially the CART model provides practical means to evaluate an individual’s test results, by simply noticing the marker value and subsequently following the branches of the tree. The MLR model on the other hand provides a probability of being a case based on methylation marker values. Therefore, this model is more generalizable to the whole population compared to the decision tree. Hence, both methods can be used depending on the requirements that follow the specific clinical demand.

A limitation of this feasibility study includes the relatively small set of samples. Also, the control group of the urine supernatant cohort included somewhat younger subjects and relatively more females than in the patient group (Table 1). Although we aimed for completely aged-matched case and control groups and succeeded in the unfractioned urine cohort, there was a small age difference in the urine supernatant cohort (median age 66 vs 60). Furthermore, no patients with CRC precursor lesions or other cancer types were included, which would better define a potential role of urine in cancer screening. In this study, markers were based on a systematic literature review on blood markers for CRC detection^3^. Genome wide methylation analysis of CRC urine specimens could possibly discover novel markers with better performance in urinary cfDNA. Furthermore, in this study, 40 ml of urine was used for DNA isolation, being a technical limitation of methodology. Other studies studying urine for cancer detection have utilized larger volumes (up to 120 ml) of urine^38, 44^. Methylation analysis with higher inputs of urine for DNA isolation might increase test accuracy and reduce test failures.

The promising results of the present study warrant further verification and validation studies, comprising larger samples series and application of pre-defined thresholds of the models used in this study. Depending on the designated clinical setting however, a validation study might require thousands of test subjects^45^. Recent large-scale efforts to screen for cancer in a population setting, have shown DNA methylation to be particularly suited for cancer detection and tissue of origin localization^46^. Nevertheless to increase test accuracy, it seems attractive to combine methylation analysis with additional molecular ctDNA markers, such as DNA mutations and/or copy number variations^47^ or with the analysis of CRC-associated metabolites^48^.

In conclusion, this study demonstrates the feasibility of urine supernatant for detection of CRC. Through means of DNA methylation analysis of a marker panel consisting of *SEPT9* and *SDC2*, CRC could be detected with high accuracy. This is the first step in conceiving a urine-based test for CRC and ultimately, other cancers as well.

## Supplementary Information

Supplementary Information.

## Data Availability

The data generated and analyzed during the current study are available from the corresponding author on reasonable request.

## References

[1] Bray F (2018). Global cancer statistics 2018: GLOBOCAN estimates of incidence and mortality worldwide for 36 cancers in 185 countries. CA Cancer J. Clin..

[2] Siegel RL (2017). Colorectal cancer statistics, 2017. CA Cancer J. Clin..

[3] Bach S (2019). Circulating tumor DNA analysis: clinical implications for colorectal cancer patients. A Systematic Review. JNCI Cancer Spect..

[4] Issa IA, Noureddine M (2017). Colorectal cancer screening: an updated review of the available options. World J. Gastroenterol..

[5] Rex DK (2017). Colorectal cancer screening: recommendations for physicians and patients from the U.S. Multi-Society Task Force on Colorectal Cancer. Am. J. Gastroenterol..

[6] Palmer CK, Thomas MC, von Wagner C, Raine R (2014). Reasons for non-uptake and subsequent participation in the NHS Bowel Cancer Screening Programme: a qualitative study. Br. J. Cancer.

[7] Vermeer NC (2017). Colorectal cancer screening: systematic review of screen-related morbidity and mortality. Cancer Treat. Rev..

[8] DeStefanis RA, Kratz JD, Emmerich PB, Deming DA (2019). Targeted therapy in metastatic colorectal cancer: current standards and novel agents in review. Curr. Colorectal. Cancer Rep..

[9] Piawah S, Venook AP (2019). Targeted therapy for colorectal cancer metastases: a review of current methods of molecularly targeted therapy and the use of tumor biomarkers in the treatment of metastatic colorectal cancer. Cancer.

[10] Normanno N, Cervantes A, Ciardiello F, De Luca A, Pinto C (2018). The liquid biopsy in the management of colorectal cancer patients: current applications and future scenarios. Cancer Treat. Rev..

[11] Thierry AR, El Messaoudi S, Gahan PB, Anker P, Stroun M (2016). Origins, structures, and functions of circulating DNA in oncology. Cancer Metastasis Rev..

[12] Laird PW, Jaenisch R (1996). The role of DNA methylation in cancer genetic and epigenetics. Annu. Rev. Genet..

[13] Luczak MW, Jagodzinski PP (2006). The role of DNA methylation in cancer development. Folia Histochem. Cytobiol..

[14] Diehl F (2008). Circulating mutant DNA to assess tumor dynamics. Nat. Med..

[15] Ma Z, Williams M, Cheng YY, Leung WK (2019). Roles of methylated DNA biomarkers in patients with colorectal cancer. Dis. Mark..

[16] Rasmussen SL (2016). Hypermethylated DNA as a biomarker for colorectal cancer: a systematic review. Colorectal Dis..

[17] Botezatu I (2000). Genetic analysis of DNA excreted in urine: a new approach for detecting specific genomic DNA sequences from cells dying in an organism. Clin. Chem..

[18] Salvi S (2016). The potential use of urine cell free DNA as a marker for cancer. Expert Rev. Mol. Diagn..

[19] Bryzgunova OE, Laktionov PP (2015). Extracellular nucleic acids in urine: sources, structure, diagnostic potential. Acta Naturae.

[20] Melkonyan HS (2008). Transrenal nucleic acids: from proof of principle to clinical tests. Ann. N. Y. Acad. Sci..

[21] Su YH (2008). Removal of high-molecular-weight DNA by carboxylated magnetic beads enhances the detection of mutated K-ras DNA in urine. Ann. N. Y. Acad. Sci..

[22] Su YH (2004). Human urine contains small, 150 to 250 nucleotide-sized, soluble DNA derived from the circulation and may be useful in the detection of colorectal cancer. J. Mol. Diagn..

[23] Hentschel AE (2020). Comparative analysis of urine fractions for optimal bladder cancer detection using DNA methylation markers. Cancers (Basel).

[24] Bosschieter J (2018). A protocol for urine collection and storage prior to DNA methylation analysis. PLoS ONE.

[25] Warren JD (2011). Septin 9 methylated DNA is a sensitive and specific blood test for colorectal cancer. BMC Med..

[26] He Q (2010). Development of a multiplex MethyLight assay for the detection of multigene methylation in human colorectal cancer. Cancer Genet. Cytogenet..

[27] Oh T (2013). Genome-wide identification and validation of a novel methylation biomarker, SDC2, for blood-based detection of colorectal cancer. J. Mol. Diagn..

[28] Song BP (2012). Detection of hypermethylated vimentin in urine of patients with colorectal cancer. J. Mol. Diagn..

[29] Xiao W (2015). Quantitative detection of methylated NDRG4 gene as a candidate biomarker for diagnosis of colorectal cancer. Oncol. Lett..

[30] Snellenberg S (2012). Development of a multiplex methylation-specific PCR as candidate triage test for women with an HPV-positive cervical scrape. BMC Cancer.

[31] Breiman L, Friedman JH, Olshen RA, Stone CJ (2017). Classification and Regression Trees - 1st Edition.

[32] Bosschieter J (2019). A two-gene methylation signature for the diagnosis of bladder cancer in urine. Epigenomics.

[33] Kitchener HC, Owens GL (2014). Urine testing for HPV. BMJ.

[34] Roobol MJ, Bangma CH, el Bouazzaoui S, Franken-Raab CG, Zwarthoff EC (2010). Feasibility study of screening for bladder cancer with urinary molecular markers (the BLU-P project). Urol. Oncol..

[35] Snoek BC (2019). Cervical cancer detection by DNA methylation analysis in urine. Sci. Rep..

[36] Amiot A (2014). The detection of the methylated Wif-1 gene is more accurate than a fecal occult blood test for colorectal cancer screening. PLoS ONE.

[37] Fraser HB, Lam LL, Neumann SM, Kobor MS (2012). Population-specificity of human DNA methylation. Genome Biol..

[38] Fujii T (2017). Mutation-enrichment next-generation sequencing for quantitative detection of KRAS mutations in urine cell-free DNA from patients with advanced cancers. Clin. Cancer Res..

[39] Lamb YN, Dhillon S (2017). Epi proColon((R)) 2.0 CE: a blood-based screening test for colorectal cancer. Mol. Diagn. Ther..

[40] Hausmann R (2010). Electrical forces determine glomerular permeability. J. Am. Soc. Nephrol..

[41] Beagan JJ (2020). Circulating tumor DNA as a preoperative marker of recurrence in patients with peritoneal metastases of colorectal cancer: a clinical feasibility study. J. Clin. Med..

[42] Dienstmann R (2017). Consensus molecular subtypes and the evolution of precision medicine in colorectal cancer. Nat. Rev. Cancer.

[43] Ubink I (2018). Histopathological and molecular classification of colorectal cancer and corresponding peritoneal metastases. Br. J. Surg..

[44] Reckamp KL (2016). A highly sensitive and quantitative test platform for detection of NSCLC EGFR mutations in urine and plasma. J. Thorac. Oncol..

[45] Hajian-Tilaki K (2014). Sample size estimation in diagnostic test studies of biomedical informatics. J. Biomed. Inform..

[46] Liu MC (2020). Sensitive and specific multi-cancer detection and localization using methylation signatures in cell-free DNA. Ann. Oncol..

[47] Cheng THT (2019). Noninvasive detection of bladder cancer by shallow-depth genome-wide bisulfite sequencing of urinary cell-free DNA for methylation and copy number profiling. Clin. Chem..

[48] Deng L (2019). Urinary metabolomics to identify a unique biomarker panel for detecting colorectal cancer: a multicenter study. Cancer Epidemiol. Biomark. Prev..

