# Supplementary material for: Detection of colorectal cancer in urine using DNA methylation analysis
Source: Sci Rep. 2021 Jan 27;11:2363. doi: 10.1038/s41598-021-81900-6 (PMC7840909; doi:10.1038/s41598-021-81900-6)
Supplement: Supplementary file 1 — Supplementary Information. [file 41598_2021_81900_MOESM1_ESM.pdf]

Detection of Colorectal Cancer in urine using DNA methylation analysis

S. Bach[1], I. Paulis[1], N. R. Sluiter[1], M. Tibbesma[2], I. Martin[3], M.A. van de Wiel[3], J. B. Tuynman[1], I. Bahce[4], G. Kazemier[1]*, R.D.M. Steenbergen[2]*

Supplementary S1

Full description of qMSP methylation marker calculations

Methylation marker abundance was calculated relative to ACTB levels (Ct-ratio), using the following formula: 2 - (CtMARKER – CtACTB) * 100. Samples with a β-actin Ct value >32 were considered to be of insufficient quality to allow for reliable methylation analysis. Using unfractioned urines, 2 out of 49 CRC patients were excluded based on a ACTB Ct value >32 (i.e. Ct value of 32.5 and 33), compared to none of 20 control subjects.

For urine supernatant, 14 out of initial 58 CRC samples, and 5 (multiplex 1) or 6 (multiplex 2) out of 48 control samples had a ACTB >32 and were excluded from analysis.

Samples showing no methylation (but sufficient DNA, i.e ACTB ≤32) were evaluated as having a Ctratio value of 0,0001. As a positive control, bisulfite treated RKO cell line DNA was used, with ultrapure water acting as a negative control. Detection rates, depicting the proportion of samples within a group showing methylation for any marker (Ct<45) were expressed in percentages.

Full description of statistical analysis

Data analysis

Ct-ratios of methylation targets were compared between groups using the Mann Whitney U test. Results from statistical tests were corrected for multiple testing by the Bonferroni-procedure. Differences in absolute detection rates were compared and tested for statistical significance with the Pearson's Chi-square test. P values < 0.05 were considered to be statistically significant. Detection rates, depicting the proportion of samples within a group showing methylation for any marker (Ct<45) were expressed in percentages.

Further analyses of relationships between methylation and clinical parameters were only performed for marker SEPT9, since all other markers did not have sufficient data points for additional statistical analysis. In the patient group, SEPT9 was compared to cancer stage by the Kruskal Wallis test. Due a large proportion of stage IV patients in our study having only peritoneal metastases (68%), results of stage IV patients were split up between peritoneal metastasis solely and stage IV including all types of (including haematogenous) metastasis. In this study this were only liver metastases.

To determine the ability of this urine supernatant-based qMSP marker panel to differentiate between controls and patients, two individual approaches were explored for determining both the best marker panel and marker thresholds for a maximal test accuracy.

In the first method, multivariate logistics regression was used to model the probability of a urine sample being from a CRC patient, with all six methylation markers as independent variables. We first fit the logistics model with all main effects followed by stepwise selection to obtain the most parsimonious model. The Akaike Information Criterion (AIC) was used to select markers that are associated with the probability of being a case. To investigate whether the in-model effect of an individual marker was affected by other markers, we fitted a model with interaction terms (containing the selected marker) followed by stepwise selection. A leave-one-out cross-validation was then used to evaluate the performance of the model for prediction. Next, the predicted

probability from this cross-validation was used for sample classification. We consider a threshold which maximizes Youden's index, i.e. the sum of sensitivity and specificity minus 1. The R function glm was used for the fitting of logistics regression model.

Apart from the logistics regression, we applied an algorithm-based method called classification and regression tree (CART) for binary classification of cases and controls on the same set of methylation markers. A tree was built by recursively split features with gini index used as a measurement of node impurity. The final tree was obtained by optimizing each split on maximizing purity. We refer to [1] for further details on CART method. For prediction purpose, the predicted class was obtained by leave-one-out cross validation. The R package rpart is available to build the decision tree as well as performing prediction.

The performance of both methods were quantified from the sensitivities and specificities. Specifically for logistics regression, the ROC curve was plotted together with maximized Youden's index. When comparing the results from both logistics regression model and CART, we noticed that both method support the conclusion of each other in terms of the prognosis factors.

All statistical analyses were performed using SPSS software (SPSS 22.0, IBM, Armonk, NY, USA) and R (Vienna, Austria. UR). Data visualization and construction of graphs was facilitated by GraphPad (Graphpad Prism version 8.2.1, La Jolla, CA USA).

Results

The effect of SEPT9 and SDC2 – logistic regression

When fitting the multivariate logistics regression model with main effects, only SEPT9 was significantly associated with the probability of being in the cases. The estimated parameter of SEPT9 is 0.21 (standard error of 0.05, p-value of < 0.0001). This means the increase of one unit SEPT9 will increase the odds of being in the cases of 23% (see Figure S1). Next, we fitted a logistics regression with SEPT9 and the interaction terms with other markers. The stepwise procedures selected SEPT9 and the interaction term between SEPT9 and SDC2 to be predictors that were associated with the probability of being the case (Table S1). Although the interaction term between SEPT9 and SDC2 fell short of statistical significant, it indicates how the probability of being in the cases was affected by different values of SEPT9 and SDC2. Figure S2 illustrates behavior of the estimated probability of being in the cases for various values of SEPT9 and SDC2. When the values of SEPT9 were very negative, the probability of being a case are relatively low irrespective of SDC2 levels. In the higher values of SEPT9, we notice gradual increases of predicted probabilities of being a case was affected by the values of SDC2. The increase of predicted probability for one unit increment of SEPT2 is slower in the lower values of SDC2.

CART Decision Tree

We used a tree model to classify cases and controls based on the values of each markers. The final tree was depicted in Figure 3 and the procedure to illustrate the tree building was supplied in Figure S3. As in the logistics regression, SEPT9 is the most important predictor for the classification. When the value of SEPT9 is higher or equal to -0.098, subjects were classified into cases (node 3, Figure 3). In node 3, there are 4 subjects who were misclassified. Consider the node 2 of SEPT9 lower than -0.098. When subjects has value of SEPT9 lower than -2.9, they will be classified into controls with 5 subjects were misclassified here (node 4). Finally, when the value of SEPT9 between -2.9 and -0.098, the threshold of SDC2 = -1.8 determined the controls (when SDC2 is lower than -1.8, 4 is

misclassified) and cases (when SDC2 is higher than -1.8, with 2 misclassification). With regard to this final tree, we notice that this model supports the logistics regression. The classification of cases and controls based on SDC2 which depends on the values of SEPT9 indicates the importance of interaction term between SEPT9 and SDC2.

Prediction and Performance

We used leave-one-out cross validation to evaluate the prediction performance of both logistics regression and decision tree. Using the logistics regression, we obtained the estimated probability of being in the case while the decision tree gives a class of either 0 and 1. Hence, unlike the decision tree, logistic regression allows to draw a ROC-curve (Figure 4). To compare the performances of both methods, a threshold for the predicted probability in the logistics regression is needed for classification. For this purpose, we opted to maximized Youden's index, equaling the sum of specificity and sensitivity minus 1. The performance of the prediction was then compared. Table 2 illustrates the performance measures from both models. In general, the performance of both logistics model with interaction and decision tree were almost similar with the logistics regression gives better sensitivities compared to the decision tree, whereas the latter has better specificity. To conclude, both classifiers agree for 95% samples (Figure S4 and Figure 5). Figure S4 demonstrates the methylation detection of each individual marker in supernatant urine of patients and controls for both analyses.

Table S1: The estimated parameters in the logistics regression model with interaction

	Estimate	Standard error	p-values
(Intercept)	0,66	0,28	0,02
SEPT9	0,57	0,22	0,01
SEPT9 : SDC2	0,03	0,02	0,05
**AIC**	**92,66**		

Table S1: The estimated parameters in the logistics regression model with interaction

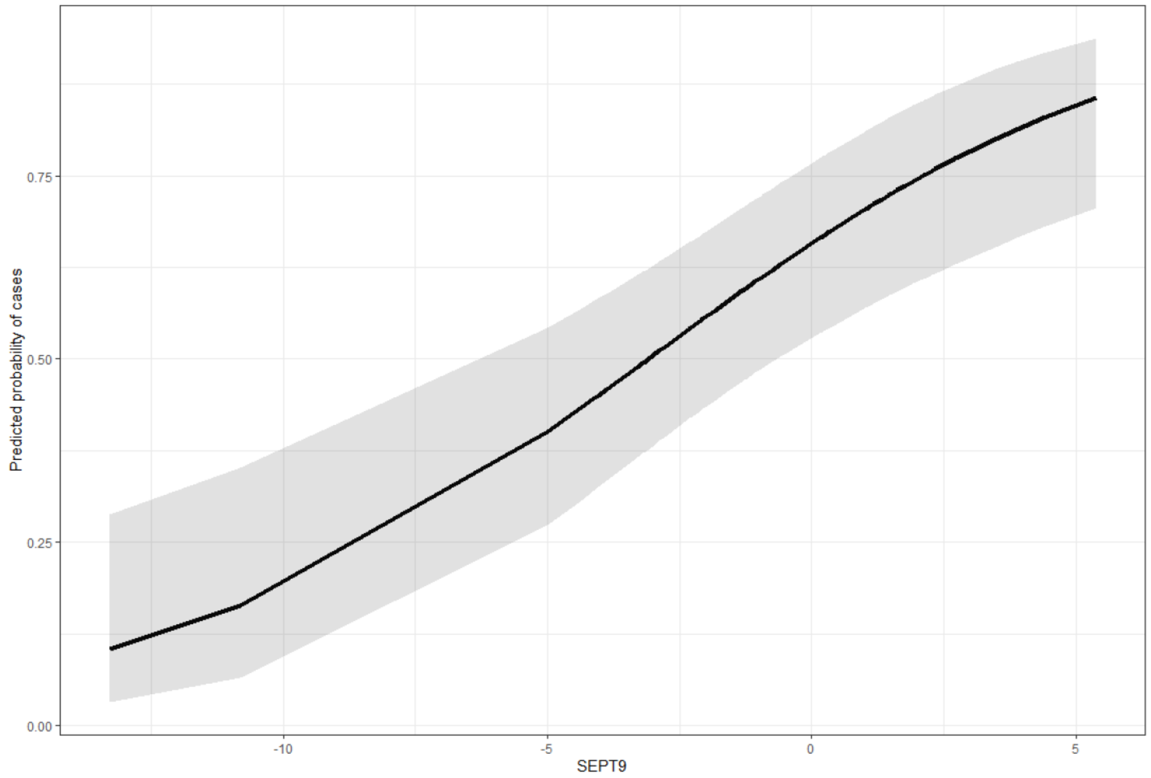

Figure S1: The predicted probability from the stepwise model from the main effect model.

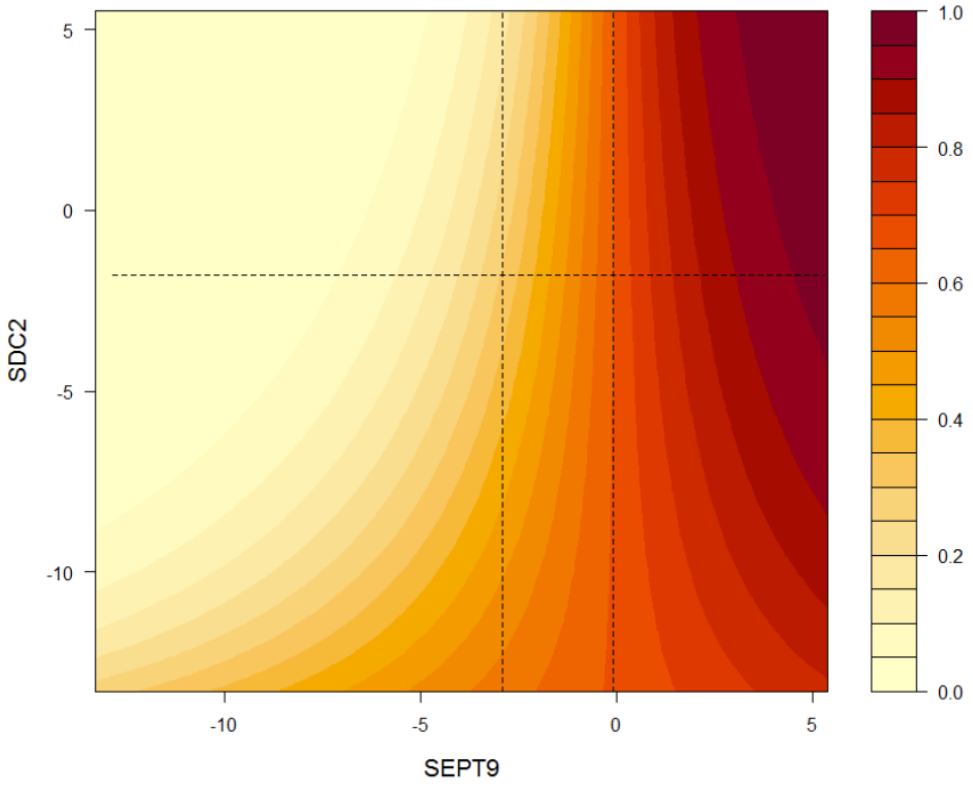

Figure S2: The predicted probability from the stepwise model from the main effect and interaction model.

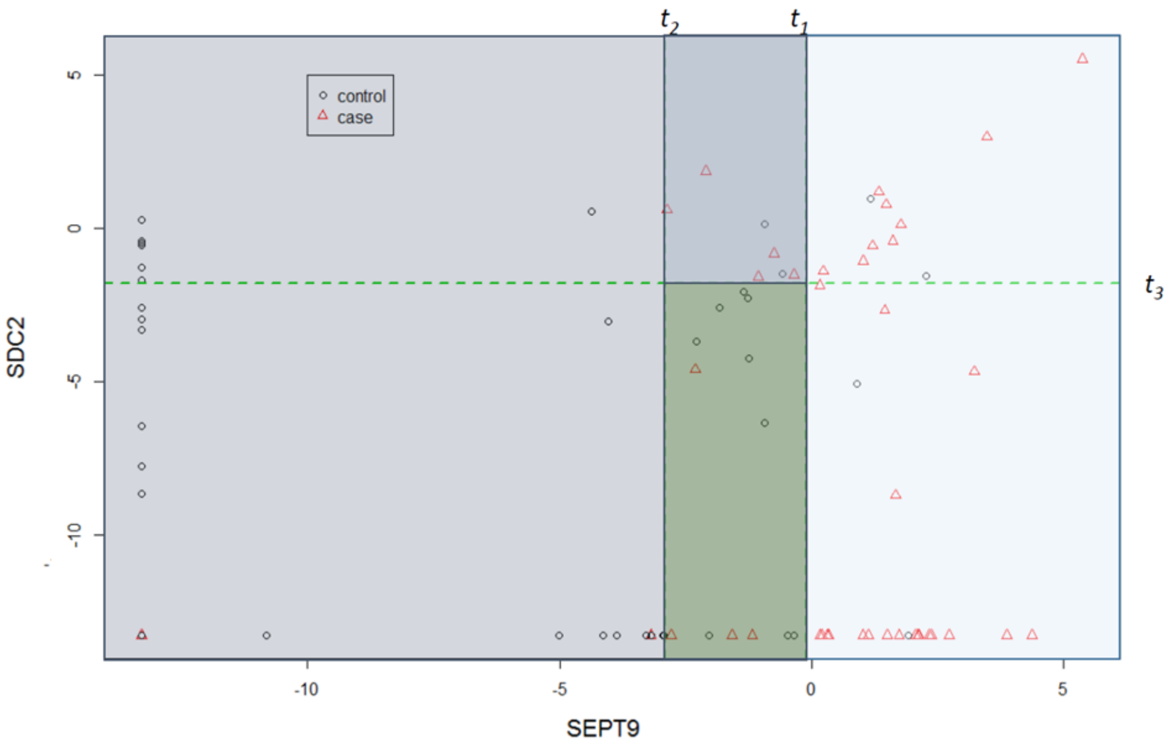

Figure S3: The illustration on building the decision tree; X-axis SEPT9 methylation ratios (log transformed), Y-axis SDC2 methylation ratios (log transformed). The triangles represent cases and circles represent controls. The dashed line represents the cutoff with t1; t2 and t2 represents the cutoff for the decision tree.

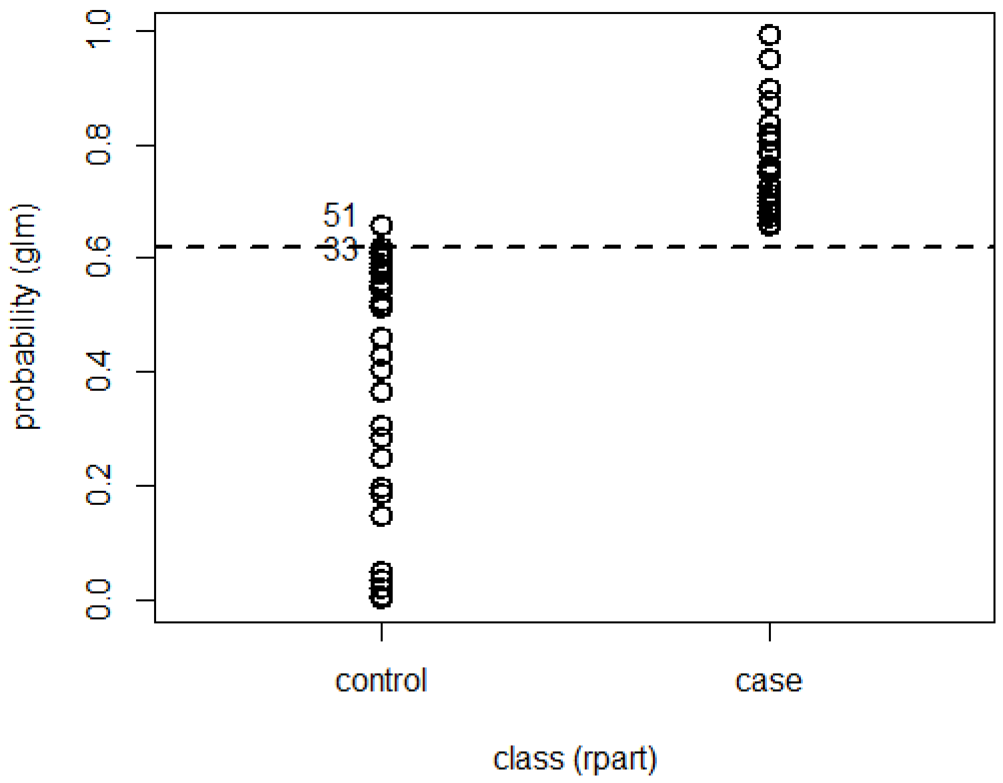

Figure S4: The predicted class from decision tree (CART; X-axis) vs the predicted probability from the multivariate logistic regression (MLR; Y-axis). The horizontal dashed line represents the threshold in which Youden's index is maximized for the prediction using multivariate logistic regression. In this figure, sample 51 and 33 were misclassified. In cases group, subject number 33 is misclassified as control by CART and in the control group, subject number 51 were misclassified as 1 by MLR.
